# Template-Assisted Preparation of Micrometric Suspended Membrane Lattices of Photoluminescent and Non-Photoluminescent Polymers by Capillarity-Driven Solvent Evaporation: Application to Microtagging

**DOI:** 10.1038/s41598-017-08278-2

**Published:** 2017-08-21

**Authors:** Giovanni Polito, Valentina Robbiano, Chiara Cozzi, Franco Cacialli, Giuseppe Barillaro

**Affiliations:** 1 Dipartimento di Ingegneria dell’Informazione, Università di Pisa, via G. Caruso 16, 56122 Pisa, Italy; 2 Department of Physics and Astronomy and London Centre for Nanotechnology, University College London, London, WC1E 6BT United Kingdom

## Abstract

In this work, the bottom-up template-assisted preparation of high-density lattices (up to 11 · 10^6^ membranes/cm^2^) of suspended polymer membranes with micrometric size (in the order of few μm^2^) and sub-micrometric thickness (in the order of hundreds of nm) is demonstrated for both photoluminescent and non-photoluminescent polymers by capillarity-driven solvent evaporation. Solvent evaporation of low concentration polymer solutions drop-cast on an array of open-ended micropipes is shown to lead to polymer membrane formation at the inlet of the micropipes thanks to capillarity. The method is proven to be robust with high-yield (>98%) over large areas (1 cm^2^) and of general validity for both conjugated and non-conjugated polymers, e.g. poly(9,9-di-*n*-octylfluorene-*alt*-benzothiadiazole (F8BT), poly[2-methoxy-5-(3′,7′-dimethyloctyloxy)-1,4-phenylenevinylene] (MDMO-PPV), polystyrene (PS), thus breaking a new ground on the controlled preparation of polymer micro and nanostructures. Angle dependence and thermal stability of photoluminescence emission arising from F8BT membrane lattices was thorough investigated, highlighting a non-Lambertian photoluminescence emission of membrane lattices with respect to F8BT films. The method is eventually successfully applied to the preparation of both photoluminescent and non-photoluminescent micro Quick Response (μQR) codes using different polymers, i.e. F8BT, MDMO-PPV, PS, thus providing micrometric-sized taggants suitable for anti-counterfeiting applications.

## Introduction

Fluids, and solvents in particular, have always played a crucial role in material conditioning for the fabrication of structures and systems at the micro and nanoscale. Liquid properties, e.g. capillarity^1^, have represented a remarkable asset for direct assembly and manipulation of materials and objects at physical scales where surface tension dominates over all other forces^2–5^. Capillarity has been also largely exploited for the self-assembly of elemental polymer objects into more complex architectures^6, 7^, although, to the best of our knowledge, its use to constrain the flow of polymer solutions through microstructured templates and, in turn, to enable the bottom-up template-assisted organization of polymer molecules into nano and microstructures upon solvent evaporation has been somehow overlooked.

A major branch of polymer technology relies on the well-known concept that the molecular structure of a polymer does not uniquely define its solid-state properties, which, on the contrary, strongly depend on the higher levels of structure arising from different processing approaches^8^. According to this, the synergistic integration of polymer technologies and basic disciplines, such as chemistry, biochemistry, engineering, and biology has generated novel functional and versatile polymer-based materials/platforms ranging from simple functionalized thin-films to highly complex architectures, with applications in nanomedicine^9, 10^, tissue culture and regeneration^11^, surface engineering^12^, bioMEMS^13, 14^, energy storage^15^, and many more.

A recent research trend of thin-film technology is directed at the fabrication of two-dimensional (2D) either free-standing or semi-free-standing polymer films in order to exploit advantages of these films over bulk polymer materials, such as larger surface-to-volume ratios and enhanced surface interactions both enabling the targeting of specific phenomena otherwise disguised by interfacial effects with the supporting substrate^16^. Free-standing magnetic nanocomposite films, self-folding polymer films for microactuation^17, 18^, and photo-switchable fluorescent films for anti-counterfeiting represent a few remarkable examples^19^. To overcome substrate-related limitations, free-standing nanometric films have been fabricated using both solid and liquid sacrificial substrates^20–25^, though dealing with critical film handling problems and mechanical instability after substrate removal. In spite of such an extensive research effort, regular pattering of suspended (i.e. not supported over a substrate) micrometric-sized polymer films/membranes has not yet been reported.

In this work, capillarity-driven solvent evaporation is exploited for the first time for the facile, versatile, and parallel bottom-up preparation of high-density lattices (from 1 to 11 · 10^6^ membranes/cm^2^) of micrometric-sized (from ~3 to ~14 μm^2^) suspended polymer membranes with sub-micrometric thickness (in the order of hundreds of nm). Template-assisted formation of polymer membranes at the inlet of 2D arrays of micrometric-sized open-ended pipes is achieved by drop-casting of low concentration (~1 wt%) polymer solutions and subsequent capillarity-driven solvent evaporation at room temperature and atmospheric pressure. The method is proven to be robust with high-yield (>98%) and of general validity for both conjugated (as illustrative examples we have tested it with poly(9,9- di-*n*-octylfluorene-*alt*-benzothiadiazole) (F8BT), and poly[2-methoxy-5-(3′,7′-dimethyloctyloxy)-1,4-phenylenevinylene] (MDMO-PPV)), and non-conjugated polymers (such as polystyrene, PS), arranged in either regular or non-regular patterns. A thorough optical characterization of F8BT membrane lattices revealed a non-Lambertian angle-dependence of the photoluminescence emission with respect to F8BT films, though coupled with comparable high thermal stability.

Finally, the potential of suspended polymer membrane lattices for microtagging applications is demonstrated by preparing photoluminescent and non-photoluminescent micro Quick Response (μQR) codes, thus providing micrometric-sized taggants suitable for anti-counterfeiting applications.

Counterfeits, in fact, generate dramatic issues in many industrial contexts, e.g. pharmaceuticals, automotive, watches, and fashion. To fight counterfeiting, several kinds of taggants, e.g. radio frequency tags, barcodes, watermarks, fluorescent inks, chemical or biological (DNA) tags, have been adopted^26^. Taggants visibility and easiness to imitate, substitute, or adulterate are just a few of the reasons driving research efforts toward the fabrication of novel, ideally covert, micrometric-sized taggants or taggants showing encoded microfeatures^27, 28^. Furthermore, conventional one-dimensional code schemes on the microtaggants provide limited features for authentication, and restoring data from a damaged code is not possible. On the contrary, the 2D dot-based binary code of QR codes^29^ combines both high-capacity encoding and the possibility of damaged code recovery, as demonstrated by micro-metric-sized QR-based taggants recently proposed^30^.

## Results and Discussion

### Preparation of High-Density Lattices of Micrometric Suspended F8BT Membranes by Capillarity-Driven Solvent Evaporation

**Figure 1:**
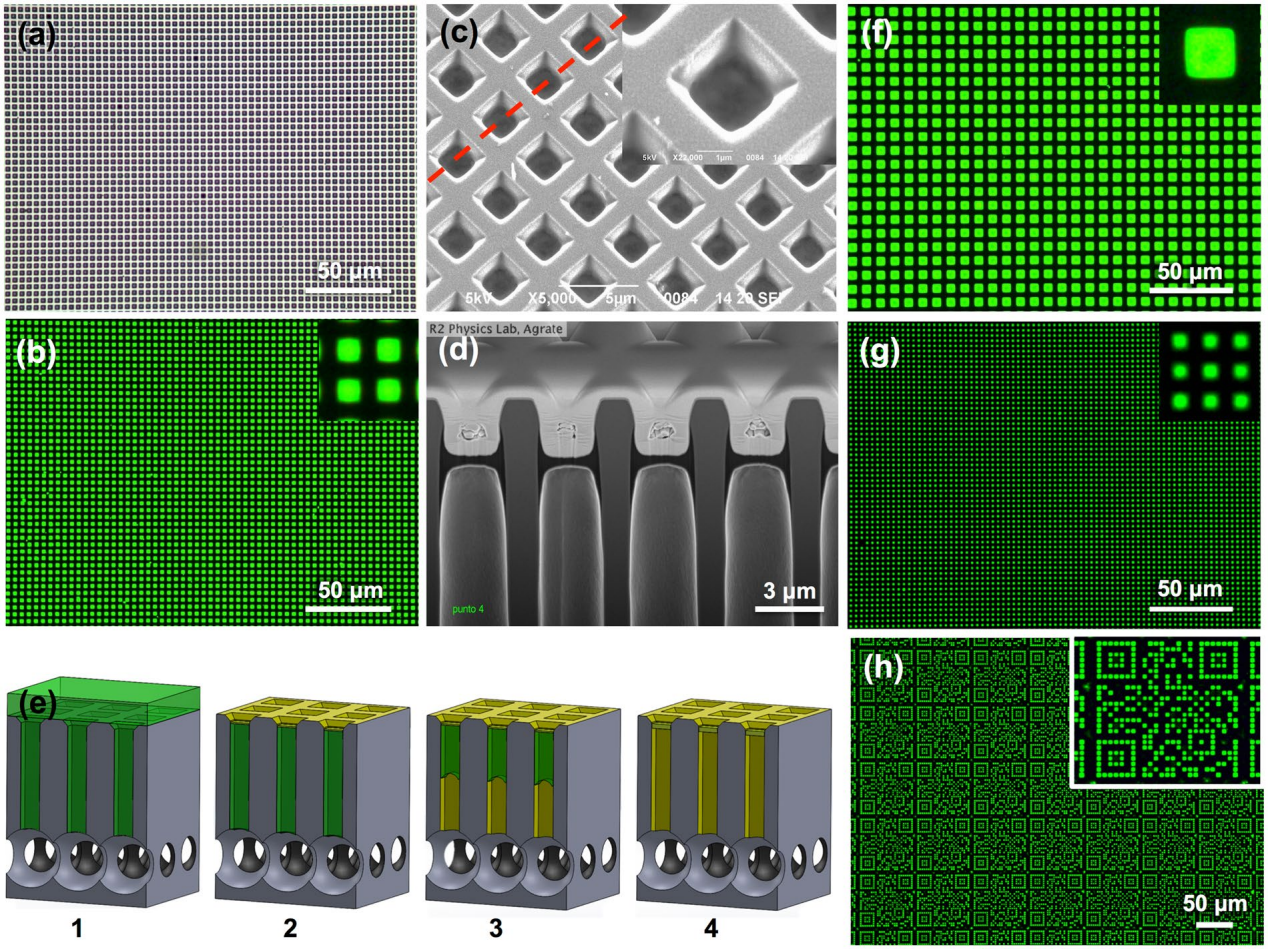
Preparation of High-Density Lattices of Micrometric Suspended F8BT Membranes by Capillarity-Driven Solvent Evaporation. (**a**,**b**,**f**,**g**) Bright-field (**a**) and fluorescence (**b**,**f**,**g**) optical images showing a top-view of suspended F8BT membrane lattices prepared drop-casting 10 μL of 1 wt% polymer solution onto silicon microstructured templates featuring 2D arrays of open-ended micropipes with pitch of 8 (**f**), 4 (**a**,**b**), and 3 μm (**g**), with insets highlighting a 10 × 10 μm^2^ area. (**c**,**d**) SEM micrographs showing a bird’s-eye view (**c**) of the membrane lattice reported in (**a**,**b**), with inset highlighting a single suspended membrane at higher magnification, and a cross-section view (**d**) obtained by FIB milling process along the red dashed line in (**c**). (**e**) Sketch showing the phases of membrane formation: (e-1) polymer filled open-ended micropipes resulting from capillarity-driven flow developing along the pipes, from inlet (top) to outlet (bottom), after drop-casting deposition, (e-2) formation of a continuous polymer layer covering the silicon microstructure upon solvent evaporation from the solution on top of the microstructure, (e-3) flow menisci moving backward upon solvent evaporation from the solution within the pipes, (e-4) suspended polymer membrane formation at the inlet of open-ended pipes after full solvent evaporation. (**h**) Fluorescence optical image showing typical μQRs of suspended F8BT membranes obtained drop-casting 5 μL of 1 wt% polymer solution, and inset reporting a zoom on a single μQR.

Figure 1a,b shows a 2D lattice of green-fluorescent suspended polymer membranes with size of ~4 μm^2^, thickness of ~300 nm, and density of 6.25 · 10^6^ membranes/cm^2^. The membranes were prepared drop-casting 10 μL of a 1 wt% F8BT^31, 32^ solution in toluene on top of a silicon microstructured template featuring a 2D array of open-ended out-of-plane micropipes with side of ~2 μm, pitch of 4 μm, and depth of 50 μm (Fig. 1e-1). Details of the template fabrication are given in the *Supplementary Information* (Fig. S1).

Evaporation of toluene at room temperature and atmospheric pressure from the micropipe array leads to the template-assisted formation of photoluminescent F8BT membranes at the pipe inlet. The suspended membranes are clearly distinguishable as bright-green squares in Fig. 1b (fluorescence images). The strong contrast with areas in which the F8BT covers the silicon microstructure is due to the quenching of F8BT photoluminescence (PL) by the silicon. A Scanning Electron Microscope (SEM) bird’s-eye view micrograph of the 2D lattice of F8BT membranes of Fig. 1a,b is reported in Fig. 1c. The membranes appear smooth and flat, uniformly plugging the pipe inlet at a depth right below the top silicon surface, which is conformably coated with the polymer. The inset in Fig. 1c shows a single F8BT membrane at higher magnification. An SEM micrograph of the cross-section of the sample in Fig. 1c, obtained by Focused Ion Beam (FIB) milling process after Chromium sputtering, is shown in Fig. 1d. Suspended membranes plugging the pipes at their inlet are clearly visible, with comparable thickness (~300 nm) and position (~1000 nm from the top). The membranes feature a double-crescent shape with a flat central region pinned to the pipe sidewalls through horn-like ends, pointing both upward and downward. A thin polymer layer with thickness of a few nanometers conformably covers the pipe inner surface.

The phases of membrane formation are sketched in Fig. 1e for the case of F8BT, though it can be assumed to be valid for different polymers as demonstrated below. After drop-casting of the polymer solution on top of the silicon microstructured template, a complete wetting regime establishes due to good wettability of organic solvents on high-energy surfaces, such as silicon^33^. All the pipes in the array are completely filled throughout their length with the polymer solution thanks to a capillarity-driven flow that develops along the open-ended pipes from their inlet (top) to outlet (bottom) and stops at the pipe outlet (bottom) where the flow menisci meet a sudden enlargement of the pipe cross-section (Fig. 1e-1). This phenomenon, which is well-known in planar microfluidics, is typical of passive capillary stop-valves where the pressure barrier that develops when the cross-section of a channel abruptly expands is exploited to stop the fluid flow^34, 35^. Here, the template was designed to have a 2D arrangement of passive capillary stop-valves operating along the out-of-plane direction, thus extending the concept from a single in-plane capillary stop-valve to an array of out-of-plane capillary stop-valves. Stop-valve operation is not affected by this out-of-plane design since in microfluidics the effects of surface tension (i.e. capillarity) are dominant over both gravitational and inertial forces, which cubically decrease with downscaling^36^. One of the major shortcomings of capillary stop-valves is the absence of a barrier preventing liquid evaporation, which becomes a concern in long-lasting experiments or if heating is part of the process^37^. Conversely, in the proposed membrane preparation process solvent evaporation represents a crucial essential step.

**Figure 2:**
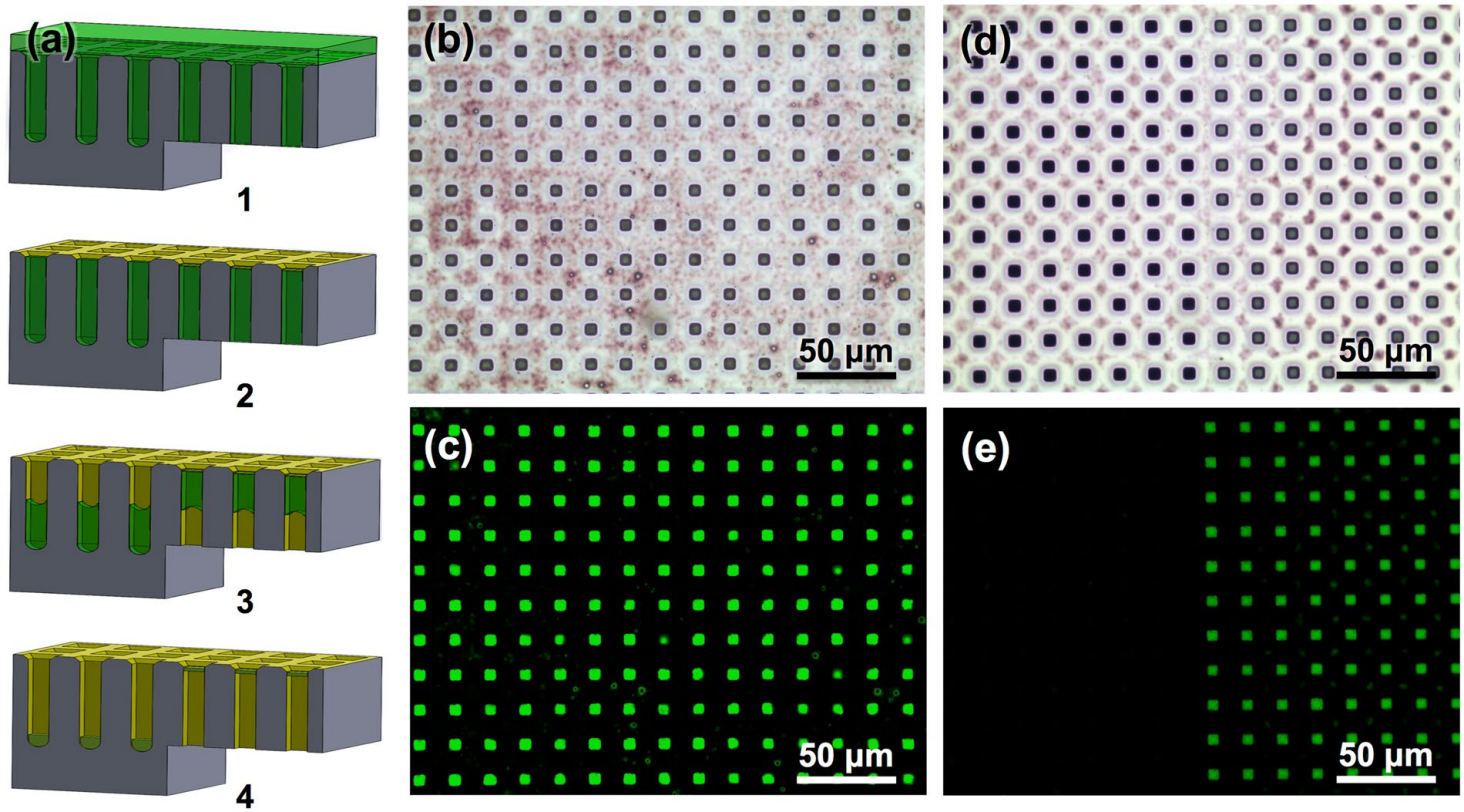
Preparation of Micrometric Suspended F8BT Membrane Lattices on Open-Ended Pass-Through Micropipes. (**a**) Sketch showing the phases of membrane formation in silicon microstructured templates featuring both closed-ended and open-ended pass-through micropipes leading to suspended membrane formation only on these latter (positive control samples): (a-1) polymer infiltration pattern resulting from drop-casting deposition of 10 μL of polymer solution with 1 wt% of F8BT onto silicon microstructured templates featuring a 2D array of both closed-ended and open-ended pass-through micropipes, also highlighting the stop-valve mechanism taking place in open-ended pass-through micropipes and the complete filling of closed-ended micropipes, (a-2) formation of a continuous polymer layer on top of the silicon microstructured template upon solvent evaporation from the solution on top of the template, (a-3) solvent evaporation causing flow menisci either to move downward in closed-ended micropipes or to move backward in open-ended pass-through micropipes, (a-4) suspended polymer membrane formation at the inlet of open-ended pass-through micropipes and thick polymer layer deposited at the bottom of closed-ended micropipes upon full solvent evaporation. (**b**–**e**) Bright-field (**b**,**d**) and fluorescence (**c**,**e**) optical top view images acquired in the area with open-ended pass-through micropipes (b,c) and at the boundary between open-ended pass-through micropipes (right) and closed-ended micropipes (left) (d,e) showing suspended F8BT membranes formed at the inlet of open-ended pass-through micropipes after drop-casting deposition of 10 μL of polymer solution with 1 wt% of F8BT onto a silicon template as sketched in (**a**).

**Figure 3:**
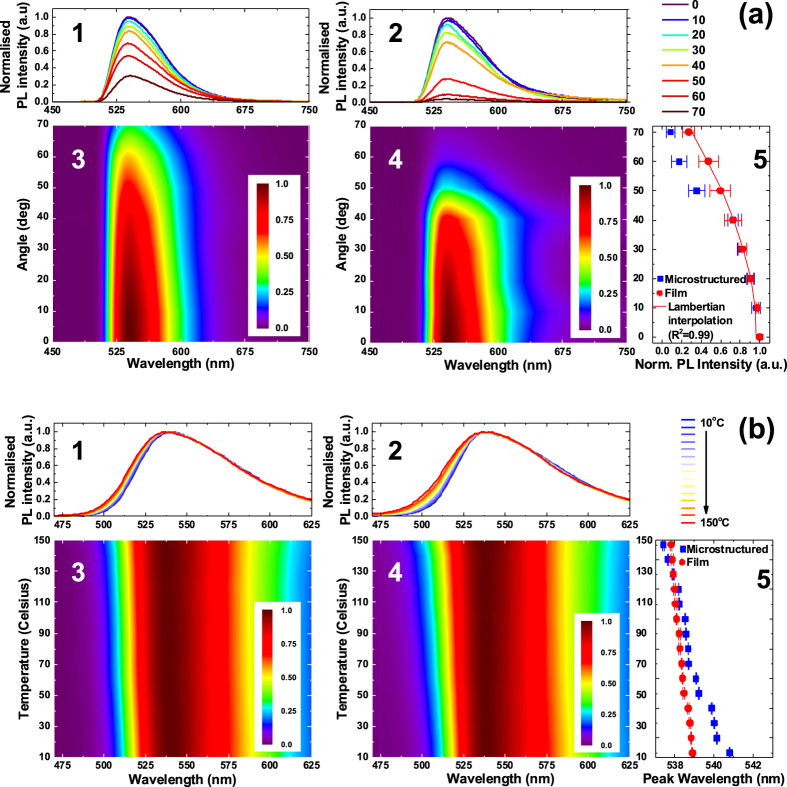
Optical Characterization of Micrometric Suspended F8BT Membrane Lattices. (**a**) PL emission as a function of the collection angle: normalized PL spectra as a function of the collection angle (a-1, a-2) and the relative contour plots (a-3, a-4) referring to F8BT suspended membrane lattices featuring pitch of 3 μm obtained drop-casting 10 μL of 1 wt% polymer solution (a-2, a-4) and F8BT films (a-1, a-3), together with a graph comparing spectrally integrated PL versus collection angle for both sets of samples (a-5). (**b**) PL emission as a function of the temperature: normalized PL spectra as a function of the temperature (b-1, b-2) and the relative contour plots (b-3, b-4) referring to F8BT suspended membrane lattices featuring pitch of 3 μm obtained drop-casting 10 μL of 1 wt% polymer solution (b-2, b-4) and F8BT films (b-1, b-3), together with a graph comparing the emission peak wavelength versus the operation temperature for both sets of samples (b-5).

**Figure 4:**
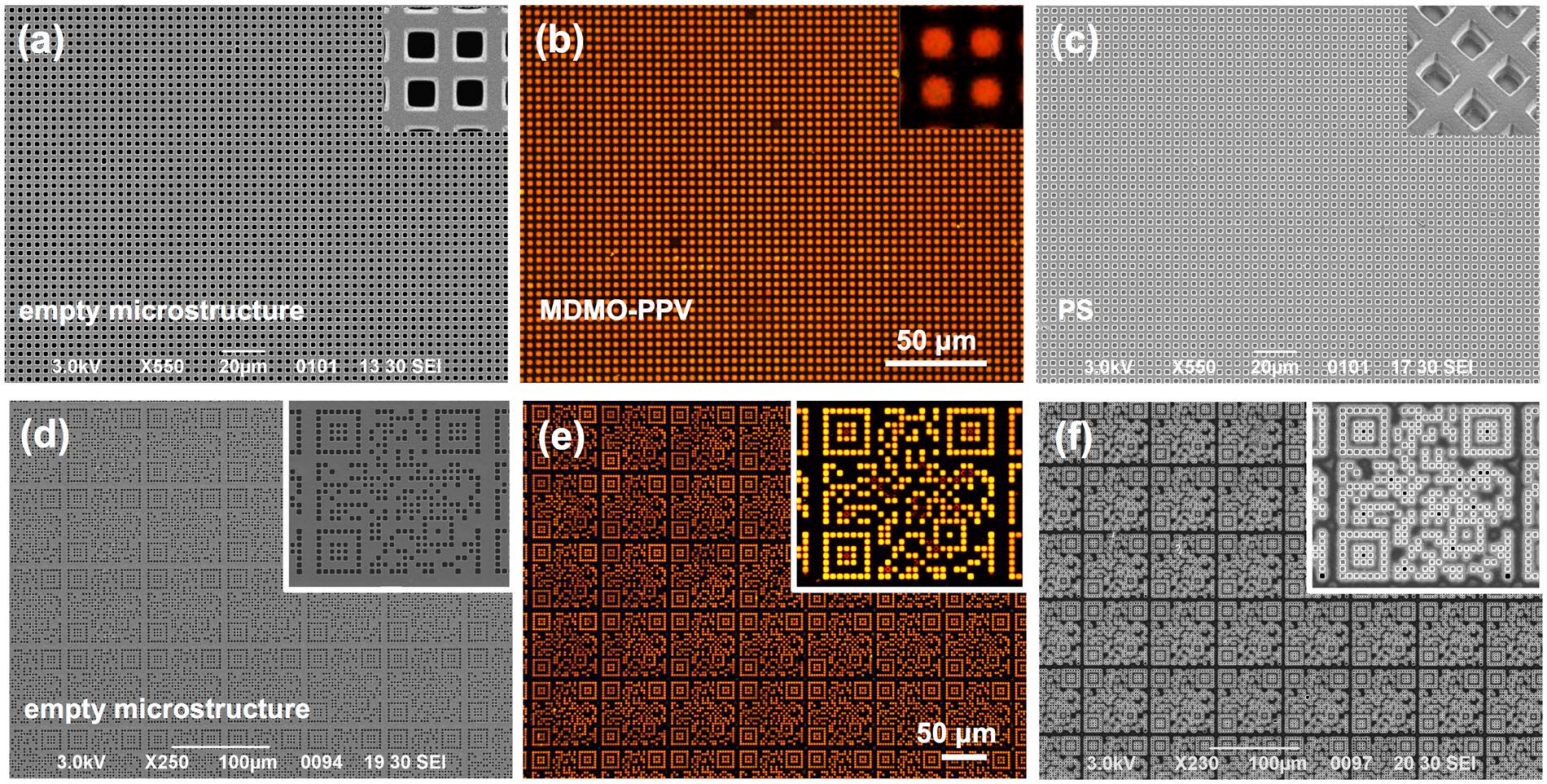
Preparation of Both Regular and Non-Regular (μQR codes) Micrometric Suspended MDMO-PPV and PS Membrane Lattices. (**a**–**c**) Top-view SEM (**a**,**c**) and 50× optical (**b**) images of a silicon template featuring 2D arrays of open-ended micropipes featuring pitch of 4 μm before polymer drop-casting (**a**) and after formation of suspended membrane lattices in MDMO-PPV (**b**) and PS (**c**), with higher magnification insets. (**d**–**f**) Top-view SEM (**d**,**f**) and 20× optical (**e**) images of a silicon microstructured template consisting of 2D repetitions of square lattices featuring a QR-code-like pattern of 2-μm-sized open-ended micropipes before polymer drop-casting (**d**) and after the formation of photoluminescent and non-photoluminescent μQRs of suspended MDMO-PPV (**e**) and PS (**f**) membranes, respectively, with higher magnification insets.

The pipe array total volume (~1 μL) is significantly smaller than the drop-cast volume, and most of the solution covers the top of the silicon template after drop-casting (Fig. 1e-1). The liquid-air interface representing the exchange surface available for solvent evaporation is, on the one hand, continuous and as wide as the whole micromachined area on top of the template, on the other hand, it consists of a 2D array of menisci pinned to the outlet (bottom) of open-ended pipes (Fig. 1e-1). Due to the larger extent of the liquid-air interface on top of the microstructure with respect to the bottom, solvent evaporation occurs at higher rate on the former. This causes randomly oriented polymer chains to densely pack and form a continuous polymer layer covering the microstructured surface, once toluene has fully evaporated from the top (yellow layer in Fig. 1e-2). At this stage evaporation of the solution left inside the pipes is allowed from both outlets (bottom) and inlets (top), occurring in the latter through the dense meshes of polymer chains plugging, partially at least, the pipe inlet. The evaporation rate is now higher at the bottom and makes the flow menisci move backward, from the outlet to the inlet of the pipes, leaving a polymer coating on the pipe walls (Fig. 1e-3). As the solvent evaporation proceeds, the concentration of polymer in the solution left inside the pipes increases and, eventually, full solvent evaporation leads to the formation of a suspended polymer membrane at the inlets of open-ended pipes, thus revealing a 2D lattice of polymer membranes (Fig. 1e-4).

The pivotal element for the template-assisted formation of suspended polymer membranes turns out to be the capillary stop-valve. Both negative (array of closed-ended pipes, Fig. S1a-3) and positive (array integrating both open-ended pass-through pipes and closed-ended pipes, Fig. S1a-4ii) control experiments were performed to corroborate this claim. Pipes in control experiments had similar size, pitch, and length of those in Fig. 1 and were drop-cast with the same (volume and concentration) F8BT polymer solutions. As for the negative control, solvent evaporation does not lead to any membrane formation. In fact, in closed-ended pipes solvent is only allowed to evaporate from the inlet (top) through the polymer chain mesh, so that the flow menisci of the polymer solution gradually move from top to bottom. Eventually, full solvent evaporation leads to the formation of a thick polymer layer at the bottom of closed-ended pipes. The phases of solvent evaporation during negative control experiments are sketched in Fig. S2a. Figure S2b–e shows optical microscope top-view and cross-section images both in bright-field (Fig. S2b,c, respectively) and in fluorescence (Fig. S2d,e, respectively) of a closed-ended pipe array after F8BT drop-casting and subsequent solvent evaporation. No suspended polymer membranes are visible from top-view images, as also pointed out by lack of fluorescence, whereas a thick polymer layer is left at the bottom of closed-ended pipes as visible from cross-section images. As for the positive control, solvent evaporation leads to the formation of a lattice of suspended polymer membranes only at the inlet of open-ended pass-through pipes. The phases of membrane formation during positive control experiments are sketched in Fig. 2a. Figure 2b,c shows both bright-field (Fig. 2b) and fluorescence (Fig. 2c) optical microscope top-view images of a 2D array of suspended F8BT membranes formed at the inlet of open-ended pass-through pipes. Figure 2d,e shows both bright-field (Fig. 2d) and fluorescence (Fig. 2e) optical microscope top-view images of the boundary region between open-ended pass-through (right-side) and closed-ended (left-side) pipes. As already observed in negative control experiments, no membrane formation (Fig. 2d, left-side) and, as a consequence, no fluorescence emission (Fig. 2e, left-side) are visible in closed-ended pipes integrated in the silicon template next to open-ended pass-through pipes.

The formation of suspended polymer membranes also using templates featuring pass-through pipe arrays, which are fully-open at their bottom, allows possible effects of the silicon substrate underneath the pipes to be ruled out, at the same time extending the field of application of the proposed preparation method, e.g. to filtration and lighting^38, 39^.

### Characterization of High-Density Lattices of Micrometric Suspended F8BT Membranes

Flexibility, reliability, and yield of the membrane formation process is investigated by preparing arrays of suspended F8BT membranes with different density and membrane size, using silicon microstructured templates featuring 2D arrays of open-ended pipes with pitch ranging from 8 to 3 μm (Fig. S1b). The drop-casting of 10 μL of 1 wt% F8BT in toluene and the following solvent evaporation result in 2D lattices of photoluminescent polymer membranes with a difference of about one order of magnitude (i.e. a factor 7) in both density, from 1.5 · 10^6^ to 11 · 10^6^ membranes/cm^2^, and single membrane size, from ~14 to ~3 μm^2^. Fluorescence images of suspended F8BT membrane lattices (at magnifications of 50× in Fig. 1b,f,g and 20× in Fig. S3a–c) show high uniformity of the preparation process over large areas, regardless of density and size of the polymer membranes. Insets in Fig. 1b,f,g show details of the suspended F8BT membranes over an area of 10 × 10 μm^2^. Notably, PL emission appears well localized in correspondence of the F8BT membranes, with high signal-to-noise ratio (S/N ~255 in pixel level ratio) owing to the fact that the polymer emission on silicon is fully quenched by the silicon. The yield of the preparation process approaches 100%, evaluated as the number of membranes formed with respect to number of pipes available over an area of 900 × 900 μm^2^ and calculated over 3 different samples (average values and standard deviation (sd) 97 ± 2%, 96 ± 4%, and 98 ± 1% for lattices with pitch of 8, 4, and 3 μm, respectively).

Further experiments were carried out on F8BT membrane lattices with a pitch of 3 μm, which have smaller membrane size and higher membrane density, to investigate possible effects of different drop-cast volumes on the preparation process. Besides 10 μL, volumes of 20 μL and 5 μL of the polymer solution were tested. Noteworthy, the preparation process is confirmed to be highly effective both increasing and decreasing the drop-cast volume, resulting in a preparation yield of ~100% membranes formed over an area of 900 × 900 μm^2^ and calculated over 3 different samples. Figure S3d–f shows fluorescence images at 50× magnifications of F8BT membrane lattices formed drop-casting different volumes of polymer solution on silicon microstructured templates featuring micropipe arrays with pitch of 3 μm.

A thorough optical characterization was performed on F8BT membrane lattices with pitch of 3 μm formed drop-casting 10 μL of polymer solution, with the aim of investigating PL emission versus both collection angle (from 0° to 70°) and operation temperature (from 10 °C to 150 °C). A 300-nm-thick F8BT film drop-cast on flat silicon was used as control. Figure 3a shows normalized PL spectra as a function of collection angle (Fig. 3a-1,-2) and the relative contour plots (Fig. 3a-3,-4) of films (Fig. 3a-1,-3) and membrane lattices (Fig. 3a-2,-4), together with a graph comparing spectrally integrated PL values versus collection angle for both films and membrane lattices (Fig. 3a-5). No obvious difference in the line shape of PL spectra is appreciable between films and membrane lattices. However, a significant and sudden drop of light intensity collected at angles ≥50° is evident for membrane lattices with respect to films by comparison of both colour contour plot and PL spectra. The vestigial pyramid-shaped features at the inlet of pipes, which are 54.7° angled with respect to the silicon wafer surface^40^, and are due to the potassium hydroxide (KOH) etching step during the template fabrication (Fig. S1a-2), shade light emitted beyond 50°. Accordingly, whereas spectrally integrated PL arising from F8BT films (red dots) is best-fitted (R^2^ = 0.99) with a Lambertian function (red line), that arising from membrane lattices (blue squares) deviates from the Lambertian law at collection angles greater than 40°. Therefore, whereas the preparation of high-density F8BT membrane lattices does not affect the F8BT emission line-shape, it turns out to be a suitable tool to engineer light emission distribution towards the fabrication, for example, of highly-directional light sources^41^.

The effect of temperature on the F8BT emission properties of both films and membrane lattices are summarized in Fig. 3b. From left to right, Fig. 3b shows normalized PL spectra as a function of operation temperature (Fig. 3b-1,-2), and the relative contour plots (Fig. 3b-3,-4) of films (Fig. 3b-1,-3) and membrane lattices (Fig. 3b-2,-4), together with a graph comparing the emission peak wavelength versus the operation temperature for both films and membrane lattices (Fig. 3b-5). From PL spectra and contour plots it can be noticed that the emission tends to blue-shift and broadens as the temperature increases, in agreement with the literature^42^.

This broadening is more pronounced for membrane lattices rather than for films. In both cases, a blue shift of the emission peak with temperature is appreciable^43^, which is, again, more significant for membrane lattices (4 nm) than for films (<1 nm). The blue-shift provides a clear indication of the reduction of average conjugation length in the material, as a result of the population of higher-energy vibrational levels, and the observation of a difference between films and membranes suggests that the material is less closely-packed in the latter, with subsequent larger free volume, and the subsequent (greater) ease for vibrational modes to be excited, with the chains being driven in a less-conjugated (average) configuration. Interestingly, despite suspended polymer membranes with sub-micrometric thickness might look fragile and more subject to thermal instability if compared to films of same thickness, we have not observed significant changes in the emission properties as function of temperature over the explored interval (10 °C–150 °C).

### Application of Capillarity-Driven Solvent Evaporation to the Preparation of Suspended Membrane Lattices Using Both Conjugated (MDMO-PPV) and Non-Conjugated (PS) Polymers

Besides F8BT, other photoluminescent and non-photoluminescent polymers, such as MDMO-PPV and PS, were successfully used for the preparation of suspended membrane lattices. MDMO-PPV is a conjugated polymer widely employed for the fabrication of organic light-emitting diodes (OLEDs)^44^, whereas PS is one of most used thermoplastic polymers employed for applications ranging from packaging and food containers^45^, to insulating foams^46^, and polymer-bonded explosives^47^. Toluene solutions with polymer concentration of ~1 wt% were prepared using both the polymers and drop-cast (10 μL) on a silicon template featuring a 2D array of open-ended pipes with pitch of 4 μm and depth of 50 μm. Solvent evaporation leads to the formation of both MDMO-PPV and PS membranes with ~100% yield, as for F8BT. This demonstrates that the preparation process is of general application to both conjugated and non-conjugated polymers, thus drastically broadening the fields of application of the proposed approach.

Figure 4 shows top-view SEM and optical images at 50× magnifications of the silicon microstructured template before polymer drop-casting (empty microstructure, Fig. 4a) and after the formation of MDMO-PPV (Fig. 4b) and PS (Fig. 4c) membrane lattices. The insets in Fig. 4b and c allow a better appreciation of MDMO-PPV and PS membranes, respectively. Remarkably, all membrane lattices show high uniformity regardless of the polymer used, in terms of both membrane formation and, for the luminescent polymer, light emission. We argue that, since different polymer solutions were prepared using the same low-surface-tension solvent (i.e. toluene), no particular alterations either in terms of silicon wetting conditions or in terms of solvent evaporation rate arose from the use of different polymers, at least in the range of molecular weights and concentrations investigated.

### Preparation of Photoluminescent and Non-Photoluminescent Micro QRs (μQRs) for Microtagging Applications by Capillarity-Driven Solvent Evaporation

Eventually, we used our approach for the preparation of photoluminescent and non-photoluminescent micro QRs (μQRs) for microtagging applications. This also allows us to demonstrate that the proposed approach is suitable for the fabrication of non-regular 2D arrays of suspended polymer membranes.

Silicon microstructured templates consisting of a 2D repetition of 2 μm-sized square holes arranged in an 81 μm × 81 μm lattice featuring a QR-code-like pattern coding for the University of Pisa website (https://www.unipi.it) were designed and fabricated so as to obtain an array of open-ended pipes, as described in Fig. S1a-1,-2,-3,-4i. A SEM top-view micrograph of the silicon microstructure is shown in Fig. 4d, with inset highlighting a single μQR code. A volume of 5 μL of polymer solution containing ~1 wt% of polymer (i.e. F8BT, MDMO-PPV, and PS) in toluene was drop-cast on top of the silicon template and let evaporate at room temperature. Figures 1h and 4e show top-view fluorescence images at 20× magnifications of μQRs in F8BT and MDMO-PPV, respectively, while μQRs in PS are shown in the SEM top-view image reported in Fig. 4f. Insets allow a better appreciation of the quality of a single μQR prepared with each kind of polymer. Regardless of the polymer used, polymer membrane formation is achieved despite the non-regularity of the pattern. In fact, the non-regularity of the pattern could lead to large flat silicon areas between micropipes that might affect solvent evaporation and, in turn, polymer distribution on top of silicon between adjacent pipes. Nonetheless, the membrane formation process seems to be not significantly affected, at least for the pattern tested in this work.

The μQR pattern is well-formed and detectable from open source QR Code Readers, thanks to the code intrinsic robustness, in spite of a few missing membranes. The location of the defects in the μQRs appears to be similar between different samples and regardless of the polymer used. We argue that missing membranes in the μQR codes identify isolated closed-ended pipes within the network of open-ended pipes, highlighting a pattern-related localization of defects affecting μQR template fabrication with the microstructuring technique used in this work. Despite that, QR code decodings were achieved with an efficiency of ~80% for both F8BT and MDMO-PPV μQR arrays by reading fluorescence microscope images collected at 50× magnifications with a QR Code Reader application on a smartphone.

## Conclusions

High-density lattices (up to 11 · 10^6^ membranes/cm^2^) of micrometric-sized (in the order of few μm^2^) suspended polymer membranes with sub-micrometric thickness (in the order of hundreds of nm) are prepared using both conjugated photoluminescent (i.e. F8BT, MDMO-PPV) and non-conjugated (i.e. PS) polymers by capillarity-driven solvent evaporation. The membrane formation occurs at the inlet of open-ended micropipes upon solvent evaporation after drop-casting of low concentration polymer solutions. High-yield (>98%) over large areas (1 cm^2^) is obtained in terms of membrane formation, thus paving the way towards the controlled and autonomous preparation of polymer micro and nanostructures. Optical characterization of the suspended F8BT membrane lattices highlights a non-Lambertian angle dependence of the integrated photoluminescence coupled with a good thermal stability.

As an example of application, micro Quick Response (μQR) codes made up of suspended polymer membranes were successfully prepared using both conjugated photoluminescent and non-conjugated polymers, i.e. F8BT, MDMO-PPV,and PS.

## Experimental Section

### Materials and Chemicals

Silicon wafers with (100) orientation, *n*-type doping, resistivity of 3–8 Ω cm, covered by a thermally-grown silicon dioxide layer with thickness of 200 nm, were provided by STMicroelectronics (Milan, Italy). Hydrofluoric acid (HF) 48 wt%, pentane (CH_3_(CH_2_)_3_CH_3_) 99 wt%, acetone (CH_3_COCH_3_) 99 wt%, 2-Propanol ((CH_3_)_2_CHOH) 99.8 wt%, toluene (C_6_H_5_CH_3_) Chromasolv® for HPLC 99.9 wt% (0.864 g ml^−1^), F8BT (Mn 10000–20000), MDMO-PPV (Mn 120000), and PS (Mw 2000000) were purchased from Sigma-Aldrich. Sodium Lauryl Sulphate (SLS) powder (CH_3_(CH_2_)_11_OSO_3_Na) was purchased from Carlo Erba Reagents. KOH pure powder at 85%, and ethanol (CH_3_CH_2_OH) 99.8 wt% were purchased from Fluka Analytical. Ammonium fluoride solution (NH_4_F) 40 wt% was purchased from Riedel-De Haën (Aldrich).

### Fabrication of silicon microstructured templates

Silicon templates featuring 2D regular arrays of open-ended micropipes differing by pipe side (1.6 μm, 2 μm and 4 μm) and pitch (3 μm, 4 μm and 8 μm) were fabricated for the optimization of the preparation process of suspended polymer membranes. Silicon templates featuring 2D regular arrays of closed-ended pipes and 2D regular arrays integrating both closed-ended and open-ended pass-through pipes with similar characteristics were fabricated for negative and positive control experiments, respectively, aimed at the validation of the membrane formation process. Silicon templates featuring 2D repetitions of non-regular arrays of open-ended pipes with side of 2 μm and pitch of 3 μm reproducing a specific QR code were also fabricated for the preparation of photoluminescent (and non) 2D microtaggants. In all cases, the depth of the pipes is 50 μm.

The layout of the silicon templates featuring regular arrays of micropipes was designed as a 2D lattice of square elements, differing by square side (1.5 μm, 2 μm and 4 μm) and pitch (3 μm, 4 μm and 8 μm). The layout of silicon templates featuring non-regular arrays of micropipes was designed as a 2D repetition of 2 μm-sized square elements arranged in an 81 μm × 81 μm 2D lattice with a QR-code-like pattern coding for the University of Pisa website (https://www.unipi.it). The template layout was patterned by standard photolithography on a photoresist layer spun on top of square slabs (2 × 2 cm^2^) cut from the silicon wafer. The pattern was replicated into the silicon dioxide layer by buffered HF (BHF) etching, performed at room temperature with a solution whose composition, by weight, is 6.8% of HF, 34.6% of NH_4_F and 58.6% of water (Fig. S1a-1). An array of inverted pyramid-shaped defects was etched through the silicon dioxide mask into the silicon surface by KOH etching, performed at 50 °C using a 20 wt% KOH solution, saturated with 2-Propanol to increase the etching uniformity (Fig. S1a-2). The defect pattern was anisotropically deep-etched into the bulk material up to a depth of 50 μm by back-side illumination electrochemical etching (BIEE)^48, 49^ using a 5 vol% HF: 95 vol% H_2_O solution, with 1000 ppm of SLS as a wetting agent, thus obtaining closed-ended micropipes used as negative control samples (Fig. S1a-3). Open-ended pass-through micropipes used as positive control samples were obtained from closed-ended micropipes by KOH silicon etch of the sample back-side through a square windows (5 mm × 5 mm) obtained by standard photolithography (Fig. S1a-4ii)^40^. Open-ended micropipes were obtained from closed-ended micropipes by switching the electrochemical etching from the anisotropic to the isotropic regime at a depth of 50 μm, thus allowing the connection of the pipes at their bottom through a port-hole 2D mesh (Fig. S1a-4i).

The anisotropic etching of the layout patterned on the silicon surface was carried out by controlling the etching current for an etching time of 2000 s. Regular arrays of micropipes featuring a pitch of 3 and 4 μm were anisotropically etched setting the etching current (*I*_*etch*_) value to 12.5 mA and decreasing it with a slope of 0.9 μA s^−1^ during the etching, whereas, those featuring a pitch of 8 μm were etched setting the *I*_*etch*_ value to 20 mA and decreasing it with a slope of 1.44 μA s^−1^. Non-regular arrays (i.e. μQR codes) were etched setting the *I*_*etch*_ value to 9.46 mA and decreasing it with a slope of 0.68 μA s^−1^ during the etching. The isotropic etching of deep-etched micropipes was performed abruptly increasing the *I*_*etch*_ value of 15 mA after the anisotropic etching was ended (i.e. after 2000 s), for both non-regular and regular arrays. The *I*_*etch*_ was then kept constant for 105, 115, 220, and 215 s for regular arrays featuring a pitch of 3, 4, and 8 μm, and for μQRs, respectively. For all microstructured templates, the etching voltage *V*_*etch*_ was kept at the constant value of 3 V during both anisotropic and isotropic etching. The etching area was a circular-shaped area of 0.64 cm^2^ (r = 0.45 cm) for all microstructured templates.

### Preparation of suspended polymer membranes

Suspended polymer membrane preparation was carried out dispensing a prescribed volume of polymer solution onto silicon microstructured templates by means of drop-casting/slow solvent evaporation technique. Three different polymers were tested, namely F8BT, MDMO-PPV, and PS. Toluene is the solvent used for all polymer solution preparation, and polymer solution composition was 1 wt% F8BT, 1.5 wt% MDMO-PPV, and 1 wt% PS, respectively. After stirring for 360 min at room temperature, F8BT and PS polymer solutions were filtered (PTFE filters, 0.2 μm) and then further stirred for 90 min before use. The filtering step was not performed for MDMO-PPV polymer solutions. Silicon microstructured templates were cleaned before polymer deposition through 300 s bath in 2-Propanol, acetone and pentane, respectively, followed by oven drying at 60 °C. Suspended membrane preparation was performed drop-casting 10 μL of polymer solution onto the whole circular-shaped etched area of the three different silicon microstructured templates featuring 2D regular arrays, then samples were left overnight in air atmosphere at room temperature for complete solvent evaporation. Different volumes of F8BT solution, namely 5 μL and 20 μL, were also tested with microstructured templates featuring the smallest pitch investigated in this work (i.e. 3 μm). The same approach for membrane preparation was also tested on microstructured templates featuring 2D non-regular arrays, performing drop-casting depositions of 5 μL of F8BT, MDMO-PPV, and PS polymer solutions, respectively.

### Characterization of silicon microstructured templates and polymer membranes

#### Scanning Electron Microscopy

SEM top-view and bird’s-eye view images, of both bare silicon microstructured templates and polymer membrane lattices, were acquired at a working power of 3 kV, using a Jeol JSM-6390 scanning electron microscope. Cross-sections of silicon microstructured templates integrating polymer membrane lattices were obtained by FIB milling of samples after Chromium sputtering, and SEM images were acquired at a working power of 3 kV using STMicroelectronics facilities.

#### Fluorescence Microscopy

Top-view fluorescence images of F8BT and MDMO-PPV 2D (both regular and non-regular) membrane lattices were collected using a standard optical microscope (Leica DM2500 M) equipped with a filter cube for green fluorescence signal acquisition. Full-frame (1536 pxl × 2048 pxl) 8-bit fluorescence images were collected with the same acquisition parameters, namely maximum excitation-light intensity (100 W) and integration time of 88.2 ms and 400 ms, for F8BT and MDMO-PPV membranes, respectively. In this way, the whole image dynamic range was exploited, with all images of F8BT membranes sharing the same maximum intensity value, thus ensuring consistency of the following statistical analysis.

#### Post-processing of F8BT membrane fluorescence images

Gwyddion data analysis software was used to statistically infer on the effectiveness of membrane preparation process. In particular, the *Grain Analysis* feature was used to process 50× fluorescence images acquired after each polymer deposition experiment, in order to count, after thresholding, the number of membranes showing a fluorescence signal whose intensity was higher than 50% of the image dynamic range. Average value and standard deviation of the number of such membranes formed over an area of 900 × 900 μm^2^ were finally evaluated using a set of four images per sample and testing the preparation process three times at each given pitch (i.e. 3 μm, 4 μm, and 8 μm).

#### Photoluminescence characterization

PL emission arising from a set of three F8BT membrane lattices, obtained by drop-casting 10 μL of polymer solution onto silicon templates featuring 2D regular arrays of micropipes with pitch of 3 μm, was compared to the emission arising from F8BT films drop-cast on flat silicon (both angular and thermal characterization). A volume of 100 μL of polymer solution was drop-cast on flat silicon and let dry in air atmosphere, thus obtaining a film featuring a thickness of ∼300 nm, fully comparable with membranes thickness. PL spectra were collected through a multimode optical fibre (600 μm core, N.A. 0.39) at distance of 5 mm from the sample using an Ocean Optics spectrometer (S2000 + VIS + NIR + ES) in combination with a 405 nm CW laser diode (Thorlabs) as excitation source. As to the angular characterization, five spectra were collected each 10° from a single investigation spot, rotating the collection probe from 0° (normal incidence) to 70° (angle resolution of 1°), while keeping the excitation source focused on the sample at a fixed position. For each angle, all collected spectra were averaged, normalized by the spectrum collected at normal incidence, and finally integrated. Temperature dependence of membrane emission was investigated keeping the collection probe at normal incidence and collecting five spectra every 10 °C, starting from 10 °C and increasing temperature up to 150 °C (temperature resolution of 0.01 °C), after a thermalization time of 5 min. For each temperature investigated, all collected spectra were averaged, normalized, and a Gaussian fitting was performed in order to evaluate the peak wavelength. Temperature was set and controlled using a Peltier-Pt100-heath sink-fan home-made system.

#### Supplementary Information

Supplementary Information reports additional experimental results about silicon micropipe arrays fabrication as well as about polymer suspended membrane lattices preparation and characterization.

## Electronic supplementary material

Supplementary Info
