# Supplementary material for: Template-Assisted Preparation of Micrometric Suspended Membrane Lattices of Photoluminescent and Non-Photoluminescent Polymers by Capillarity-Driven Solvent Evaporation: Application to Microtagging
Source: Sci Rep. 2017 Aug 21;7:8351. doi: 10.1038/s41598-017-08278-2 (PMC5566329; doi:10.1038/s41598-017-08278-2)
Supplement: Supplementary file 1 — Supplementary Info [file 41598_2017_8278_MOESM1_ESM.pdf]

Supplementary Information

Template-Assisted Preparation of Micrometric Suspended Membrane Lattices of Photoluminescent and Non-Photoluminescent Polymers by Capillarity-Driven Solvent Evaporation: Application to Microtagging

Giovanni Polito[1], Valentina Robbiano[1], Chiara Cozzi[1], F. Cacialli[2], and Giuseppe Barillaro[1]*

[1] Dipartimento di Ingegneria dell'Informazione, Università di Pisa, via G. Caruso 16, 56122 Pisa, Italy

[2] Department of Physics and Astronomy and London Centre for Nanotechnology, University College London, WC1E 6BT, United Kingdom.

*Correspondence to g.barillaro@iet.unipi.it

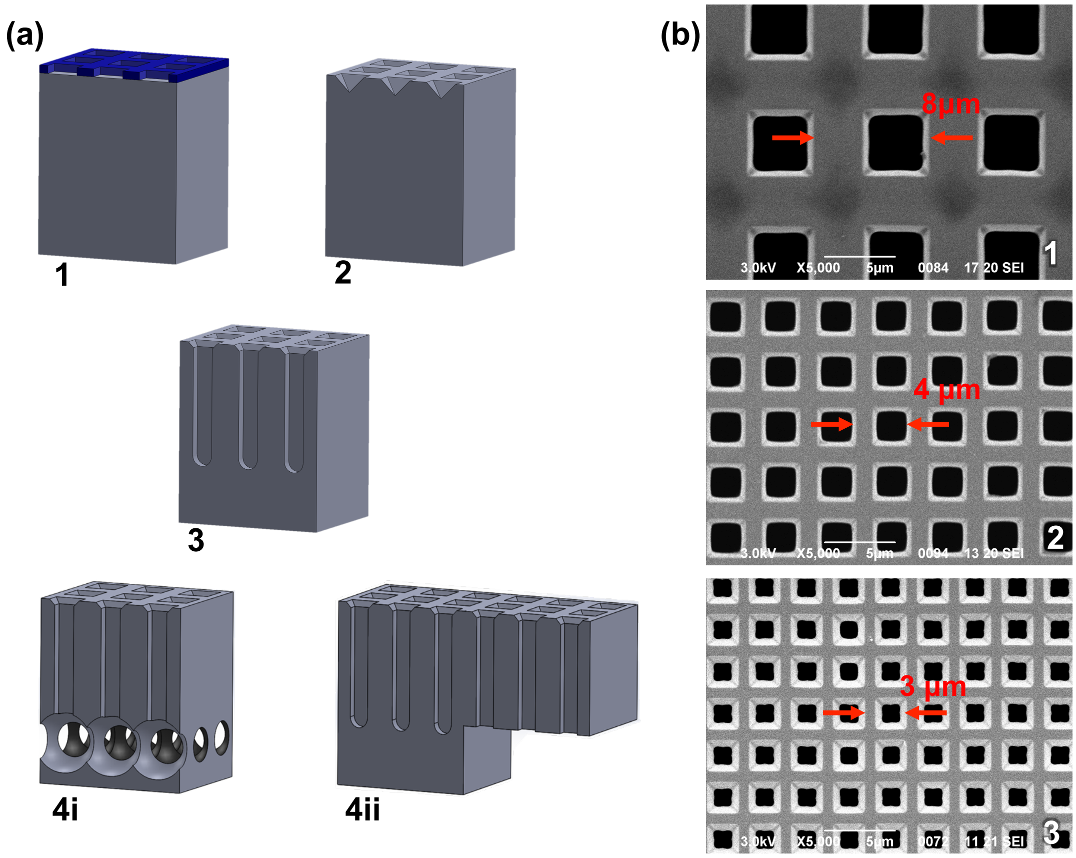

Figure S1. Fabrication of Arrays of Silicon Micropipes (a) Sketch of the main technological steps for the fabrication of 2D arrays of both regular and non regular closed-ended (a-3), open-ended (a-4i), and open-ended pass-through (a-4ii) micropipes: (a-1) definition of the layout on the silicon dioxide layer by standard photolithography and subsequent BHF etching, (a-2) layout replication into the silicon surface by KOH etching, (a-3) anisotropic deep-etch of closed-ended micropipes by BIEE (used as negative control samples), either (a-4i) switching of the etching regime from anisotropic to isotropic in order to obtain open-ended micropipes connected at their bottom by a port-hole 2D mesh or (a-4ii) KOH silicon etch of the sample back-side through a square window in order to obtain open-ended pass-through micropipes (used as positive control samples). (b) SEM micrographs showing a top-view of bare silicon micropipe arrays used for the development of the technique. The different arrays feature hole size and

pitch of about (b-1) ~14 μm^2 and 8 μm, (b-2) ~4 μm^2 and 4 μm, (b-3) ~3μm^2 and 3 μm, respectively.

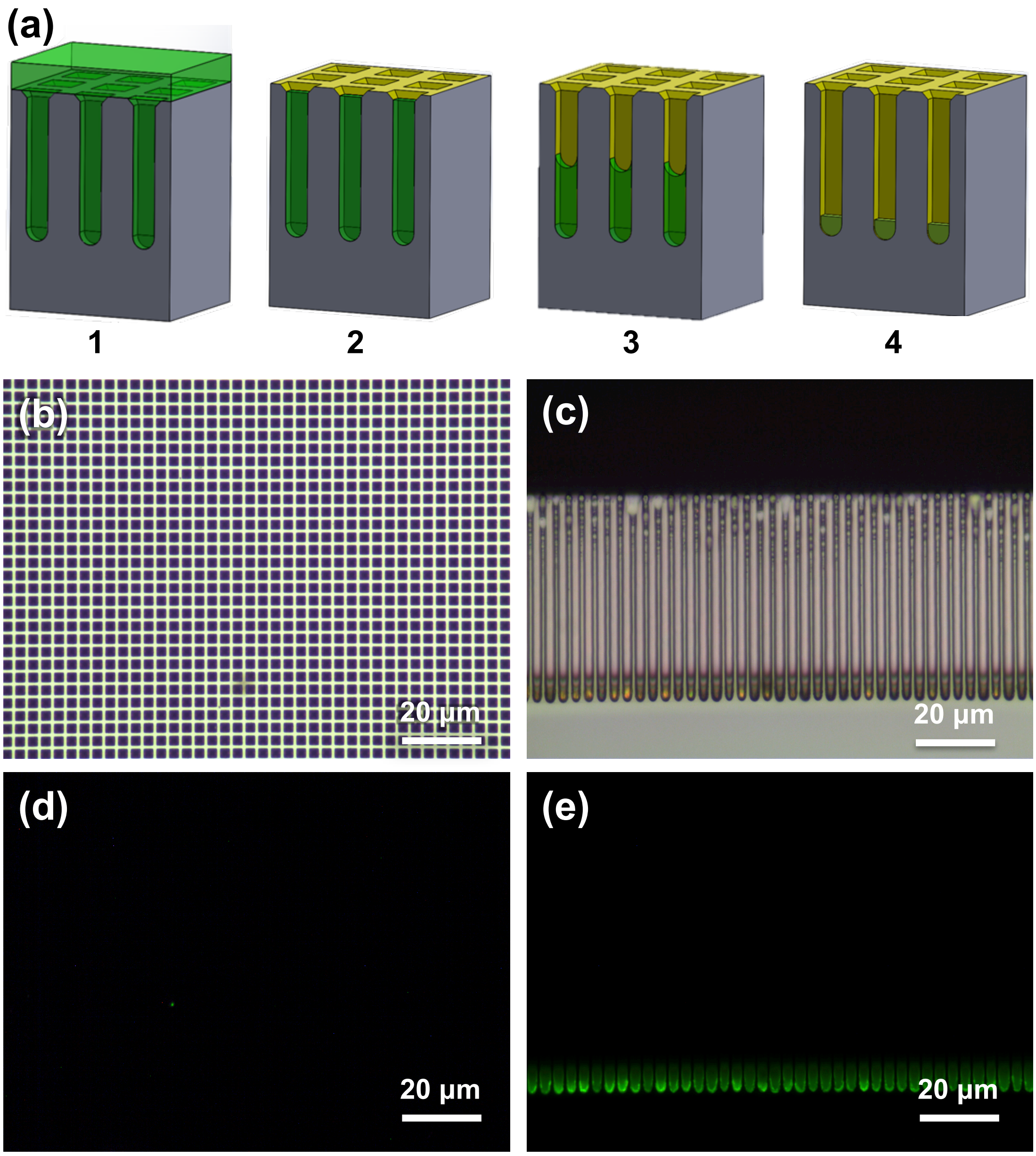

Figure S2. Closed-Ended Micropipes as Negative Control Samples (a) Sketch showing polymer infiltration and solvent evaporation in closed-ended micropipes (negative control samples): (a-1) polymer filled closed-ended micropipes resulting from drop-casting deposition, (a-2) formation of a continuous polymer layer on top of the silicon microstructured template upon solvent evaporation from the solution on top of the microstructure, (a-3) flow menisci moving from top to bottom due to solvent evaporation, (a-4) thick polymer layer left at the bottom of closed-ended micropipes upon full solvent evaporation. (b, d) Bright-field (b) and fluorescence

(d) top-view optical images showing no membrane formation occurring after drop-casting deposition of 10 μL of polymer solution with 1 wt% of F8BT onto a 2D array of closed-ended micropipes. (c, e) Bright-field (c) and fluorescence (e) optical images of a cross-section of the sample shown in (b, d) highlighting the thick polymer layer deposited at the bottom of closed-ended micropipes upon full solvent evaporation.

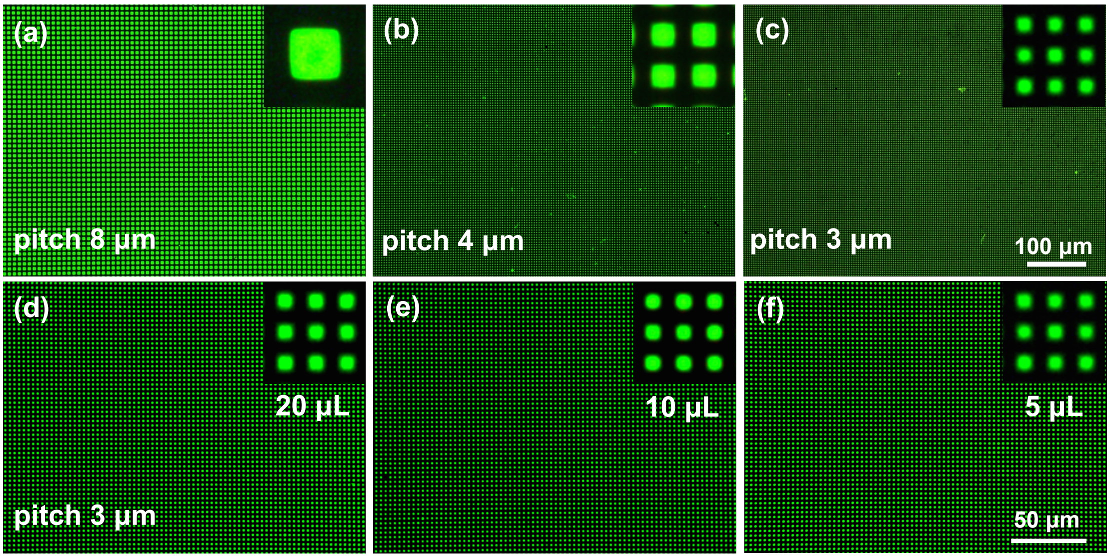

Figure S3. Flexibility of the preparation of suspended polymer membranes (a, b, c) Fluorescence images at 20× showing a top-view of 2D lattices of suspended F8BT membranes obtained by drop-casting deposition of 10 μL of polymer solution with 1 wt% F8BT onto silicon microstructured templates reported in Figure S1b-1, -2, and -3, respectively, with insets highlighting an area of 10 × 10 μm^2 of each lattice. It is possible to appreciate the effectiveness of the technique over large-area arrays. (d, e, f) Fluorescence images showing typical top-views of 2D lattices of suspended F8BT membranes obtained by drop-casting deposition of 20 μL (d), 10 μL (e), and 5 μL (f) of polymer solution with 1 wt% F8BT onto 2D open-ended micropipe arrays featuring pipe size of ~3 μm^2, and pitch of 3 μm, with insets highlighting an area of 10 × 10 μm^2 of each lattice. No evident differences were appreciable by optical and fluorescence microscopy in terms of yield and effectiveness of the method ascribable to the use of different volumes of drop-cast solution.
